# Commissioning compensator‐based IMRT on the Pinnacle treatment planning system

**DOI:** 10.1120/jacmp.v12i2.3396

**Published:** 2011-03-08

**Authors:** Daniel Opp, Kenneth Forster, Vladimir Feygelman

**Affiliations:** ^1^ Division of Radiation Oncology H. Lee Moffitt Cancer Center Tampa Florida 33612 USA

**Keywords:** Compensators, IMRT QA, diode dosimeter, 3D dosimetry, dosimeter validation

## Abstract

We present a systematic approach to commissioning of the compensator‐based IMRT in Pinnacle treatment planning system for commercially manufactured brass compensators. Some model parameters for the beams modulated by the variable‐thickness compensators can only be associated with a single compensator thickness. To intelligently choose that thickness for beam modeling, we empirically determined the most probable filter thickness occurring within the modulated portion of the compensators typically used in clinics. We demonstrated that a set of relative output factors measured with the brass slab of most probable thickness (2 cm) differs from the traditionally used open field set, and leads to improved agreement between measurements and calculations, particularly for the larger field sizes. By iteratively adjusting the modifier scatter factor and filter density, the calculated effective attenuation of the flat filters was brought to within 2% of the ion chamber measurement for the clinically‐relevant range of filter thicknesses, depths and filed sizes. Beam hardening representation in Pinnacle provides for adequate depth dose modeling beyond the depth of about 5 cm. Disagreement at shallower depth for the large field sizes is likely due to the algorithm's inability to account for the low‐energy scattered photons generated in the filter. The average ion chamber point dose error at isocenter for ten clinical compensator‐based IMRT plans was under 1%. A biplanar 3D diode dosimeter was calibrated and validated for use with the compensators. The average gamma analysis (3%/3 mm) passing rate for ten IMRT plans was 98.9%± 1.0%. The device is particularly attractive because it easily generates dose comparisons at both the fraction and beam levels. Overlaying dose profiles for individual beams would easily uncover any errors in compensator orientation.

PACS number: 87.55Qr

## I. INTRODUCTION

Pinnacle (Philips Radiation Oncology Systems, Fitchburg, WI) treatment planning system (TPS) uses a percent depth dose (PDD)‐based energy spectrum and several parameters to generate a beam model for dose calculations.^(1)^ The conceptual framework for commissioning of the Pinnacle TPS was originally presented by Starkschall et al.^(2)^ and subsequent improvements to the algorithm's ability to handle the rounded‐leaf multileaf collimator (MLC) penumbra were validated by Cadman et al.^(3)^ However, these papers address only the use of the TPS for conventional beams with traditional modifiers, such as wedges or MLC‐based IMRT. While the latter is widely accepted, there is an alternative method of two‐dimensional beam modulation – solid state compensators – which has its own advantages.^(4)^ Commissioning of the compensator‐based IMRT presents additional challenges. The compensator perturbs the beam by preferentially attenuating the lower energy photons (beam hardening effect), and by generating scattered photons and electrons.^(^ ^5^ ^–^ ^11^ ^)^ Different TPS handle (or ignore) these effects in different ways and, therefore, require a unique commissioning process. A method for commissioning brass compensator‐based IMRT has been reported for another TPS.^(6)^ The first step in this method involved acquiring a single effective attenuation coefficient for a slab of standard thickness^(12)^ and then applying a correction factor based on the actual modulator thickness. The second step involves the application of a two‐dimensional linear attenuation coefficient array to account for variability due to large field sizes.^(13)^ However, aside form brief communications,^(^ ^14^ ^,^ ^15^ ^)^ a comprehensive commissioning process for brass compensators with Pinnacle has not been described. The paper by Sasaki and Obata^(16)^ dealt with the compensators of a different design and concentrated on only one of the many variables in the beam model – the effective density of the compensator material.

Furthermore, MLC‐based IMRT QA for Pinnacle plans in our clinic is performed with a biplanar diode array dosimeter (Delta^4^, ScandiDos AB, Uppsala, Sweden). This device allows measuring and comparing of absolute three‐dimensional dose distributions. The Delta^4^ has been thoroughly evaluated by different groups for step‐and‐shoot IMRT,^(^ ^17^ ^–^ ^19^ ^)^ helical tomotherapy,^(20)^ and VMAT.^(^ ^17^ ^,^ ^21^ ^)^ It is a potentially attractive option for compensator‐based IMRT QA, as well. In addition to providing a composite dose distribution when combined with Pinnacle, the Delta^4^ workflow makes it easy to compare measured and reference doses at the beam level. It is particularly important with compensators, as the possibility of a human error in mounting a compensator is ever present. However, being a diode dosimeter, the Delta^4^ may be susceptible to the response variation with energy changes.^(^ ^22^ ^–^ ^24^ ^)^ This necessitates a separate dosimeter calibration and validation procedure for the filtered beams.

In this paper, we describe a logical method for commissioning a beam model in Pinnacle for compensators. We also describe the results of commissioning the Delta^4^ dosimeter for compensator‐based IMRT QA, thus presenting a complete planning and QA system for brass compensators.

## II. MATERIALS AND METHODS

Brass compensators were custom made by .decimal Inc., Sanford, FL. All measurements were performed with a 6 MV X‐ray beam produced by a Varian Trilogy linear accelerator (Varian Medical Systems, Palo Alto CA). Relative dose distributions were measured in the Wellhoffer Blue water tank with a CC13 0.13 cc ion chamber (IBA Dosimetry GmbH, Schwarzenbruck, Germany). Pinnacle v. 9.0 was used for treatment planning.

### A. Most probable compensator thickness

Some beam model parameters are expected to depend on the modifier thickness. However, typically the software allows only a single thickness for beam model. To obtain the best fit of measured and calculated dose across the range of realistic compensators, we determined the most probable compensator thickness. To that end, a total of 50 randomly‐selected compensators were examined. The two‐dimensional thickness matrix within the variable‐thickness region of each compensator was exported as an ASCII file and all the data were combined into a global histogram. From that distribution, the most probable compensator thickness was determined. Whenever a single modifier thickness had to be chosen for beam modeling, this most probable thickness was used.

### B. TPS parameters

Generating compensators for IMRT in Pinnacle is a three‐step process. First, an optimized fluence map is determined. It is then exported to the proprietary software (p.d, 4.1 from .decimal) that designs a compensator based on the fluence map and the effective attenuation coefficient of brass. Finally, the compensator thickness matrix is imported back into Pinnacle for the final dose calculations. If the results are acceptable, this matrix is electronically transferred to the vendor (.decimal) for manufacturing.

Pinnacle uses the superposition‐convolution algorithm, which is a model‐based method.^(2)^ The software allows the user to adjust several parameters to improve agreement between measured and calculated dose distributions. Specific parameters that may be different for brass compensator compared to the open beam model are the energy spectrum, lateral fluence, relative output factors and modifier scatter factor. The effective attenuation of the compensator material also needs to be determined.^(16)^ However, the effective attenuation coefficient is independent of the beam model and can be adjusted in the planning phase by varying the density of the compensator material.^(16)^ The methods to determine the relevant parameters for the compensator‐based beam model are discussed below.

#### B.1 PDD

The energy spectrum is adjusted during beam modeling to obtain the best fit to the measured set of the PDD curves. The algorithm accounts for the beam hardening by a physical modifier by adding its radiological thickness to the patient radiological depth along any given ray.^(1)^ The beam spectrum is depth‐dependent. The same general approach is used for physical wedges and compensators. However, there is a significant difference in its practical implementation for the different types of beam modifiers. Since the wedge shape is the same during both modeling and planning, the beam spectrum can be adjusted with the wedge in place, resulting in a separate wedge‐specific beam model. The compensators are different for every case. Therefore, it is recommended in the Pinnacle user manual^(25)^ to use the spectrum from the open beam model. To verify that the beam hardening for the compensators is handled correctly, the PDDs collected with the open beam and the beam filtered by the 3 cm thick brass slab were compared with calculations for the $5\times 5\;{\text{cm}}^{2}$ field. This combination of relatively thick filter and a small filed size is well suited for the demonstration of the beam hardening effect. Next, a set of measured PDDs for the beams filtered by a brass slab of the most probable thickness was compared to calculations for a series of field sizes from 5×5 to $20\times 20\;{\text{cm}}^{2}$. The observed dose errors were considered indicative of the central axis PDD modeling accuracy. The reported percentage dose errors are normalized to the maximum dose as described in the AAPM TG 53 report.^(26)^

#### B.2 Lateral profiles

We used the cone model to describe the lateral in‐air fluence change. The fluence increases linearly with distance from the central axis up to the maximum cone radius.^(2)^ The increase in fluence per unit distance and the radius are both adjustable parameters selected to best fit the measured lateral profile. The beam softening away from the central axis is modeled by adjusting the off‐axis softening parameter.^(2)^

It is apparent that the lateral profiles would be affected by the presence of the compensator in the beam. Therefore, a measured set of profiles with the uniform thickness brass slab in the beam was used for modeling. Since a single beam model has to cover a range of possible compensator thicknesses, the most probable brass thickness was used. To account for the beam hardening effect during modeling, the brass slab was treated as a “wedge” of uniform thickness. The parameters were adjusted to best fit a set of lateral dose profiles collected in water for a $20\times 20\;{\text{cm}}^{2}$ beam filtered by a brass slab of the most probable thickness. These parameters were then used in a separate open beam model intended for compensator‐based IMRT.

#### B.3 Output factors

For monitor unit calculations, Pinnacle requires absolute accelerator output under reference conditions for an open beam (e.g., $10\times 10\;{\text{cm}}^{2}$ field size, 10 cm depth, 90 cm source to surface distance (SSD)). It is measured directly in a water phantom. In addition, Pinnacle requires relative output factors measured for a range of field sizes ($S_{\text{cp}}$ ^(27)^), also at the reference depth. These empirical relative output factors are used to correct the calculated central axis dose at the reference depth for open beams and wedges. The compensators are treated differently from the wedges: the output factors are computed directly during the planning process as a product of the open beam output factor and the compensator attenuation factor, without a previously determined correction. Since the open field PDDs are used in the model, historically the open field output factors were used, as well. However, it is expected that the relative output factors would depend on the compensator thickness because of the variation in scatter.^(28)^ To determine the set of values providing the overall best agreement between measurements and calculations, relative output factors for a range of square filed sizes from 4×4 to $20\times 20\;{\text{cm}}^{2}$ were compared for different compensator thicknesses: 0, 1, 2, 3, and 7 cm. Output factors were measured in the $30\times 30\;{\text{cm}}^{2}$ Plastic Water phantom (CIRS Inc., Norfolk, VA) with a 0.6 cc Farmer ion chamber at the reference depth (10 cm) and 90 cm SSD.

#### B.4 Effective attenuation

Dependence of effective attenuation of .decimal compensator material on filed size and depth was studied by Bartrum et al.^(12)^ In Pinnacle, there are two parameters that can be adjusted to modify effective beam attenuation. In the planning stage, the effective attenuation of the compensating material is manipulated by changing its physical density.^(16)^ Since the compensator material density affects the spectrum of the beam incident on the phantom, its effect on the calculated effective attenuation is depth‐dependent. The second parameter affecting the effective attenuation of the filter is modifier scatter factor (MSF). It can be modified during the modeling process and is intended to account for excess scatter produced by beam modifiers such as physical wedges and compensators. The primary fluence is increased by the factor of (1+ MSF× L), where L is the modifier thickness along the ray.^(2)^ As evident from the equation, the factor is not depth or field size dependent, which is a simplification. A two‐step process was used to optimize the effective attenuation of brass. First, the compensator density was fixed at the published value for brass, and the MSF was optimized to best fit the experimental data by comparing the attenuation of the brass slabs of different thickness (1–3 cm) for different field sizes (5–20 cm), but at a single depth (10 cm). Further refinement of the effective attenuation factors was achieved by slightly varying the compensator material physical density to produce the best fit to the experimental attenuation data for the range of attenuator thicknesses and field sizes described above, but also at different depths (5–20 cm).

### C. Model validation

#### C.1 Absolute dose profiles in water for modulated beams

Dose distributions for three typical clinical compensators were calculated on a water phantom with a 3 mm dose grid resolution. Lateral profiles in the IEC X (cross‐plane) and Y (in‐plane) directions were measured in the water phantom with the CC13 ion chamber. The ion chamber readings were converted to absolute dose by comparing the readings at the point of intersection of the X and Y profiles to the reading under reference conditions: open $10\times 10\;{\text{cm}}^{2}$ field, 90 cm SSD, 10 cm depth. To minimize gradient‐related dose errors, the orthogonal profiles intersection points (scan origins) were chosen to be in the low‐dose gradient region in both X and Y directions. The water phantom SSD was 87.5 cm, to facilitate direct comparison of the longitudinal profiles with the Delta^4^ dosimeter, as described later. The orthogonal profiles were taken at 5, 12.5, and 20 cm depths. These absolute dose measurements were compared to the calculated dose profiles extracted from the TPS.

#### C.2 Point dose IMRT verification

Ten typical clinical cases were planned and the compensators were designed and manufactured in a usual manner. Consistent with our clinical practice, five cases represented pancreatic cancer, four liver, and one esophageal disease. Corresponding IMRT QA plans were created on a $20\times 20\times 20\;{\text{cm}}^{3}$ Plastic Water cube phantom (CIRS Inc., Norfolk, VA) with a 3 mm calculation grid. Measurements were performed with a 0.03 cc PTW PinPoint ion chamber (model 31015, PTW‐Freiburg, Freiburg, Germany) at isocenter for all plans. Chamber readings were converted to absolute dose by comparison to a reading under the reference conditions in the Plastic Water phantom.

#### C.3 Inhomogeneity correction

For completeness, the ability of the beam model to handle low‐density heterogeneities when a brass filter is present in the beam was validated. A Plastic Water phantom with low density wood $\left(0.27\;{g/\text{cm}}^{3}\right)$ inserts was used. Both Plastic Water and wood slabs were capable of accommodating the PinPoint chamber. No additional corrections are necessary for the chamber in a low‐density material when its atomic number is reasonably close to water.^(29)^ The geometry and measurement point positions were chosen to approximate those utilized in the AAPM TG65 report,^(29)^ to include the points both inside and beyond the inhomogeneity. The phantom consisted of 3 cm of Plastic Water followed by approximately 5 cm of low‐density wood, followed in turn by 15 cm of Plastic Water. The calculated and measured ratios of inhomogeneous to homogeneous phantom dose (correction factors) were compared. Also, the results were compared to the benchmark data quoted in the AAPM report, whenever available.

### D. Delta4 commissioning

The Delta^4^ dosimeter consists of 1069 p‐type silicone diodes 0.78 mm^2^ in size. The detectors are embedded into boards that form two orthogonal planes within a polymethyl methacrylate (PMMA) cylindrical phantom 22 cm in diameter. The “main” detector plane has an active area of $20\times 20\;{\text{cm}}^{2}$ with diodes placed 5 mm apart within a central $6\times 6\;{\text{cm}}^{2}$ region and 10 mm apart elsewhere. The second orthogonal plane is split in to two “wings” that have an area of $20\times 10\;{\text{cm}}^{2}$ each, with the same overall detector spacing. Further details on the Delta^4^ design and performance can be found in previous publications.^(^ ^17^ ^–^ ^19^ ^)^

#### D.1 Absolute calibration

Our Delta^4^ calibration procedure has been detailed previously.^(18)^ For compensators, a unique absolute calibration must be performed with a flat slab of brass of the most probable thickness in the beam. Absolute calibration was verified by comparing the Delta^4^ central dose to an independent diode detector (Isorad‐3, Sun Nuclear Corp., Melbourne, FL).^(18)^ The diode was calibrated against a Farmer chamber in the Plastic Water phantom, again with the most probable thickness of brass in the beam. An additional correction for excess scatter in PMMA was determined by intercomparison of the diode readings in PMMA and Plastic Water at the same source to detector distance (100 cm) and water‐equivalent depth (12.5 cm).^(20)^ Following the Isorad diode calibration, the main detector board was removed from the Delta^4^ phantom and replaced by the two PMMA slabs. The Isorad diode was positioned between those slabs in the middle of the phantom. This assembly was centered on the room lasers and irradiated by two $10\times 10\;{\text{cm}}^{2}$ parallel‐opposed horizontal beams. The dose readings of the six Delta^4^ diodes on the wing boards immediately surrounding the Isorad diode, were averaged and compared to the dose reported by the Isorad detector.

#### D.2 Dose distributions with flat brass filters

The effectiveness of the Delta^4^ relative calibration with an attenuator in the beam was evaluated with a single‐field and four‐field box plans. Field sizes of 10×10,15×15, and $25\times 25\;{\text{cm}}^{2}$ were used The single field used the gantry angle of 0°, while the four‐field plans used the four cardinal angles. All beams passed trough a brass filter of the most probable thickness.

It was expected that the Delta^4^ diodes would over‐respond to the low‐energy scattered photons prevalent at shallow depth. Comparison with the TPS was therefore performed in two steps. First, the level of disagreement between the TPS and measurements with an essentially energy‐independent detector (ion chamber) was established in a cylindrical phantom.

To that end, the dose was calculated on a 30 cm diameter Solid Water “Cheese” phantom (TomoTherapy Inc., Madison, WI). The relative dose in the filtered beam was measured with an Exradin A1SL 0.06 cc ion chamber (Standard Imaging Inc, Middleton, WI) in 1 cm increments along the radial line angled at 50° to the vertical, and compared to the corresponding dose profile extracted from the TPS. The measurement points thus coincided with one of the Delta^4^ detector planes. After the baseline accuracy of the calculations at different depths was established with ion chamber measurements, the experimental Delta^4^ dose distributions were compared to the TPS calculations using the γ analysis^(30)^ with the 3%/2 mm thresholds. The global dose error threshold in the Delta^4^ software was used and normalization dose therefore affected the analysis results, even though absolute dose distributions were compared.^(18)^ The dose was normalized at the isocenter for the four‐field boxes and to the maximum value for the single fields.

#### D.3 Dose profiles through isocenter for modulated beams

The Delta^4^ centered on the room lasers was exposed to the beams containing the same modulated compensators as described in the Materials Section C.1 above. The water tank Y profile at 12.5 cm depth has the same source‐to‐detector distance and water‐equivalent depth as the series of diodes on the long axis of the Delta^4^ phantom (11 cm of PMMA scaled by relative electron density of 1.14^(31)^). Therefore, the absolute dose profiles could be directly overlaid for comparison.

#### D.4 Clinical plans

The final validation step involved evaluating ten clinical compensator‐based plans. The quality assurance plans for the same ten cases used for the point dose verification were recalculated on the Delta^4^ phantom with a 3 mm dose grid. The reference dose on the fraction and beam levels was exported from Pinnacle in RTOG format to the Delta^4^ software for comparison with measurements. The legacy RTOG format was used because currently DICOM RT dose export is disabled in Pinnacle for compensator‐based plans. The gamma analysis (3%/3 mm and 2%/2 mm) results are reported. All plans were normalized to the prescription dose.

## III. RESULTS & DISCUSSION

### A. Most probable compensator thickness

**Figure 1:**
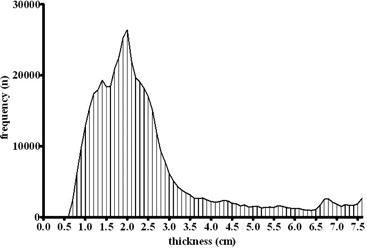
Histogram of compensator thickness; values were compiled from 50 2D thickness matrices.

The histogram in Fig. 1 shows the combined thickness frequency distribution in the modulated region of 50 typical clinical compensators. The range of modulation occurs mainly within thicknesses of 1–3 cm, with 2 cm being the most probable one. For commissioning a modified beam model for Pinnacle and calibrating the Delta^4^, the standard compensator thickness was therefore considered to be 2 cm.

### B. TPS parameters

#### B.1 PDD

**Figure 2:**
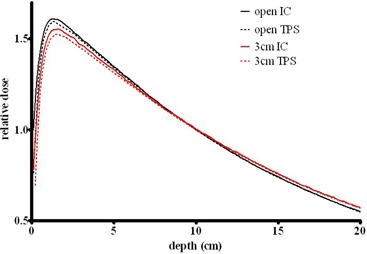
Measured (ion chamber (IC) in water, solid lines) and calculated (dashed lines) PDDs for an open 5 xxbu 5 cm^2^ beam and the same field with a 3 cm brass slab.

The depth dose curves in Fig. 2 indicate the ability of the TPS to innately accommodate beam hardening for beam modifiers. A small filed size $\left(5\times 5\;{\text{cm}}^{2}\right)$ and relatively thick brass filter (3 cm) were chosen for illustration. The depth‐dose curves are normalized at 10 cm. The largest discrepancy occurs within the $d_{\text{max}}$ region where disagreement is −1.3% for open fields, and −1.7% for fields with 3 cm brass in beam. Between the depths of 5 and 20 cm, the measured dose for the filtered beam deviates from the calculation by no more than 1.2%.

**Figure 3:**
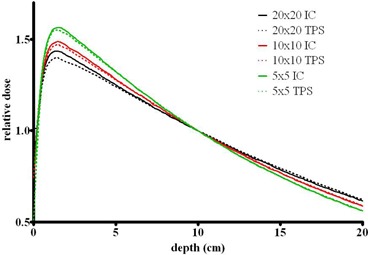
Measured (solid lines) and calculated (dashed lines) PDDs for beams filtered by 2 cm brass slab for field sizes of 5×5,10×10, and $20\times 20{\text{cm}}^{2}$.

The depth dose agreement achieved with an open beam model for the beams filtered by the most probable thickness of brass – 2 cm – is displayed in Fig. 3. The largest error again occurs at $d_{\text{max}}$ and is −3.4%, −2.7% and 1.4% for field sizes of 20×20,10×10 and $5\times 5\;{\text{cm}}^{2}$, respectively. At a depth of 5 cm and field size of $20\times 20\;{\text{cm}}^{2}$ the error is −1.5%, and for all other points the agreement is within 1%. The calculated and measured PDDs for the largest $\left(20\times 20\;{\text{cm}}^{2}\right)$ field clearly do not match well at shallow depths. This is likely due to the algorithm not accounting explicitly for the low‐energy scattered photons generated in the beam modifier. Attempts to improve the modeling by modifying the electron contamination within the planning software proved unsuccessful. This is a model limitation that has to be accepted. In our practice, compensators are typically used for relatively deep‐seated tumors.

#### B.2 Lateral profiles

**Figure 4:**
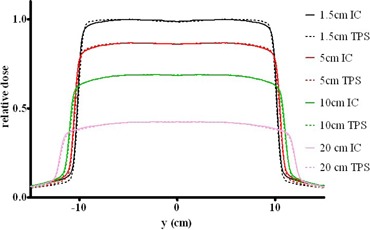
Measured (solid lines) and calculated (dashed lines) lateral dose profiles at different depths in water for a $20\times 20\;{\text{cm}}^{2}$ beam filtered with a 2 cm brass slab.

For the cone model, the fluence increase was set to $0.002{\text{cm}}^{-1}$, with a cone radius of 30 cm. The optimal softening parameter was determined to be $6.75{\text{radian}}^{-1}$. The superimposed measured and calculated relative dose profiles for a 2 cm brass filter are presented in Fig. 4. The overall dose agreement in the central 90% area of the field is better than 2%, which is satisfactory. The largest disagreement is 1.6% at the 1.5 cm depth. This is again likely due to the scattered radiation not accounted for by the beam model. It was demonstrated previously^(16)^ that penumbra width is not changed appreciably by the compensators compared to the open beam. Therefore, the set of “out‐of‐field” parameters previously determined for the open beam was used.

#### B.3 Output factors

**Figure 5:**
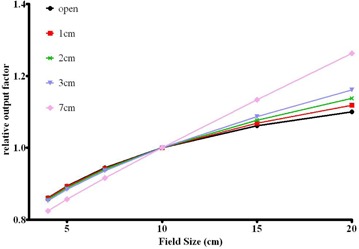
Relative output factors with varying thickness of brass in the beam: no brass (black), 1 cm (red), 2 cm (green), 3 cm (blue), and 7 cm (pink) brass slabs were evaluated.

Figure 5 illustrates the dependence of the relative output factors $\left(S_{\text{cp}}\right)$ on the brass filter thickness. For equivalent field sizes smaller than 10 cm, the output factors differ by less than 1% with the filter thickness variation from 0 to 3 cm. However, for a $20\times 20\;{\text{cm}}^{2}$ field, the relative output factor differs by 3.8% as the brass thickness changes from 0 to 2 cm. With such a difference at larger field sizes, the relative output factor table corresponding to the most probable compensator thickness (2 cm) would lead to more accurate results, and was incorporated into the beam model.

#### B.4 Effective attenuation

The optimal combination of the MSF and effective density for .decimal brass compensators was found to be $0.11{\text{cm}}^{-1}$ and $8.25\;{g/\text{cm}}^{3}$, respectively. Percentage dose errors for the attenuation factors (measured minus calculated) corresponding to this parameter selection are presented in Table 1 for a range of filter thicknesses (1–3 cm), depths (5–20 cm), and field sizes (5–20 cm). With the exception of one data point (3 cm filter, $20\times 20\;{\text{cm}}^{2}$ field, 5 cm depth), the calculations and measurements agree to within 2%.

**Table 1:** Point dose percent errors (measured – calculated) on the central axis with the optimized parameter set, for a range of depths, filed sizes and filter thicknesses.

*Brass Thickness (cm)*	Field Size (cm)	*Percent Dose Error (measured – calculated)*		
		Depth 5 cm	Depth 10 cm	*Depth 20 cm*
	5	−0.7	−1.3	−0.6
3	10	0.2	−0.5	0.5
	20	2.8	1.2	1.6
	5	0.5	−0.1	0.8
2	10	0.7	0.0	1.1
	20	1.0	−0.2	0.1
	5	1.1	0.4	1.3
1	10	0.8	0.0	1.1
	20	−0.9	−1.9	−1.4

### C. Model validation

#### C.1 Absolute dose profiles in water for modulated beams

**Figure 6:**
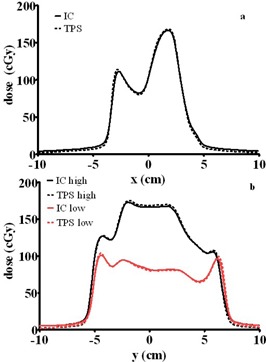
An example of orthogonal absolute dose profiles for a modulated beam at a depth of 12.5 cm and SSD of 87.5 cm, in the X (a) and Y (b) directions. The solid lines represent water phantom scans (IC) and the dotted lines corresponding calculated profiles. The Y profiles (b) were taken through the two regions of varying dose levels.

An example of overlaid measured and calculated absolute dose profiles in the X and Y directions is presented in Fig. 6. Among all three compensators, the maximum dose error outside the penumbra region did not exceed 3.2%.

#### C.2 IMRT point dose verification

The mean point dose error at isocenter for ten clinical plans was 0.8%± 0.3% (range 1.7% to −1.1%). The magnitude and standard deviation of the error distribution compares favorably with those presented in the AAPM TG119 report.^(32)^

#### C.3 Inhomogeneity correction

Measured and calculated correction factors agreed to better than 2% across all investigated depths, field size and filter thicknesses (Table 2). In addition, for the $10\times 10{\text{cm}}^{2}$ field, our calculated correction factors agreed to within 1.5% with the 6 MV data in the AAPM TG85 report.^(29)^

**Table 2:** Percent difference between measured and calculated heterogeneity correction factors for filtered beams (5×5 and $10\times 10\;{\text{cm}}^{2}$ field sizes). The column indicated by TG65 demonstrates the agreement between the TPS and the data from the AAPM TG65 report for a $10\times 10\;{\text{cm}}^{2}$ open field.

*Depth (cm)*		*Percent Difference in Correction Factors (measured – calculated)*							
	3 cm brass			2 cm brass			*1 cm brass*		
	5×5	10×10	TG65	5×5	10×10	TG65	5×5	10×10	*TG65*
4	1.3	0.3	0.4	1.2	0.4	0.6	1.5	0.7	0.8
7.3	0.3	−1.9	−1.5	0.0	−1.3	−1.0	0.2	−0.9	−0.4
9.3	−0.4	−0.9	−0.5	−0.3	−0.7	0.0	−0.3	−0.3	0.3
13.3	−0.9	−0.6	0.5	−0.7	−0.6	0.9	−0.6	−0.5	1.3

### D. Delta4 commissioning

#### D.1 Absolute calibration

The central six diodes measured an absolute dose average of 148.6±0.9 cGy while the Isorad diode positioned between them recorded 148.0 cGy. This agreement (0.4%) validates the absolute Delta^4^ calibration method with the brass slab in the beam.

#### D.2 Dose distribution with flat brass filters

Table 3 demonstrates the level of agreement between the calculated and measured (ion chamber and Delta^4^) doses at different depths along the cylinder radius. The difference in dimensions between the Cheese phantom and the Delta^4^ precludes direct comparison between the ion chamber and diode measurements. However, based on the differences between the measurements and corresponding calculations on the cylindrical phantoms (albeit of slightly different diameters), it is clear that the Delta^4^ results should fall within 2% of the ion chamber measurements at all depths. As expected, the Delta^4^ measured dose exceeds the reference value by the largest amount (2.4%) at shallow depths.

**Table 3:** Percent dose error (measured – calculated) between Pinnacle generated doses and the respective measurement methods (Ion Chamber and ${\text{Delta}}^{4}$), for a 2 cm brass filter in a $25\times 25\;{\text{cm}}^{2}$ field. The gantry is vertical. The depths are reported from the surface of the phantom along the radius at 50° angle to vertical (${\text{Delta}}^{4}$ main detector board). Ion chamber measurements were performed in a cylindrical Cheese phantom rotated to match the ${\text{Delta}}^{4}$ main board angulation.

	*Dose error (measured – calculated) (%)*	
*Depth (cm)*	Ion Chamber	${\text{Delta}}^{4}$
1	0.6	2.3
2	0.5	2.4
3	0.6	1.1
4	0.8	1.9
5	1.0	2.1
6	1.2	0.8
7	1.5	1.0
8	1.6	1.1
10	1.0	−0.9
12	0.9	−0.5
14	0.0	1.2

The gamma analysis results for the simple beam arrangements with a flat 2 cm brass filter (Table 4) are adequate, with the lowest passing rate for γ (3%/2 mm) above 96%.

**Table 4:** Gamma analysis (3%/2 mm) for beams of different field sizes filtered by a 2 cm brass slab, for single fields and four‐field boxes.

*Gantry Angle (°)*	Field Size (cm)	γ(3/2 m)≤1(%)
	10×10	100.0
0	15×15	96.4
	25×25	97.8
	10×10	99.8
0+90+180+270	15×15	97.4
	25×25	99.9

#### D.3 Dose profiles through isocenter for modulated beams

**Figure 7:**
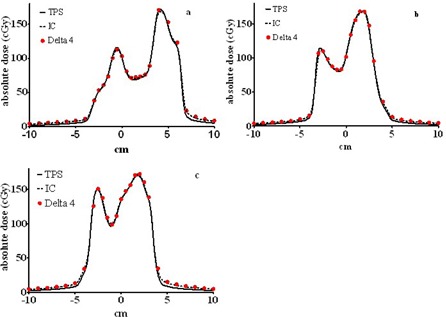
Overlaid calculated and measured (ion chamber in water and Delta^4^) absolute dose profiles in the Y direction through the isocenter for three different compensators (a, b and c). All profiles correspond to the radiological depth of 12.5 cm.

Figure 7 demonstrates overlaid water tank and Delta^4^ absolute dose profiles in the Y direction, through the isocenter, for three separate modulated beams. The worst disagreement between the ion chamber and the Delta^4^ diodes occurs in Fig. 7(a), where a few data points are about 2% or 2 mm apart. The rest of the data show better agreement. We conclude that the Delta^4^ detectors situated in the middle of the phantom show acceptable agreement with the ion chamber measurements for the typical modulated compensating filters.

#### D.4 Clinical plans

**Figure 8:**
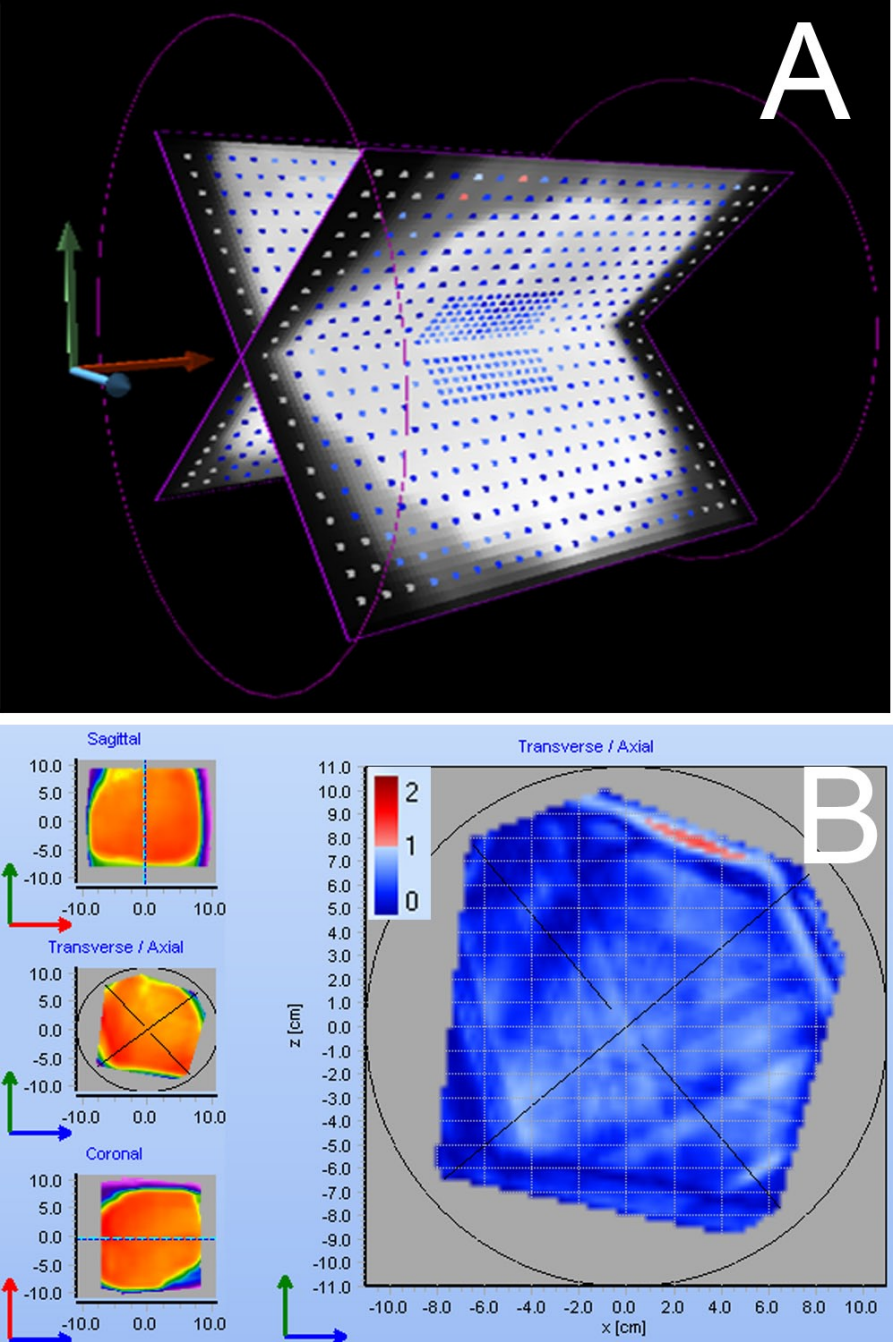
A screen shot of graphical data representation in the Delta^4^ software for a pancreatic plan: detector boards with the points passing gamma analysis in blue (a); an axial slice through the reconstructed 3D dose distribution, with areas in blue passing the γ analysis (b)

The Delta^4^ validation process was finalized by comparing the measured and calculated doses for a set of typical IMRT plans. An example of graphical data representation is shown in Fig. 8. Ten compensator‐based IMRT plans (described in Methods Section C.2) were recalculated on the Delta^4^ phantom with a 3 mm dose grid. The γ (3%/3 mm) analysis, which has become a de facto standard,^(^ ^32^ ^,^ ^33^ ^)^ revealed an average passing rate of 98.9%± 1.0% (range 97.0%–99.9%). The more discriminating 2%/2 mm thresholds result in an average gamma passing rate of 91.8%± 3.8% (range 85.1% to 96.6%). In combination with the device calibration validation by different methods and excellent agreement for independent IMRT point dose measurements presented above, as well as previous thorough validation of the Delta^4^ for MLC‐based IMRT,^(^ ^17^ ^–^ ^20^ ^)^ these results suggest that the device can be used as a routine dosimetry QA tool for compensator‐based IMRT.

## IV. CONCLUSIONS

We presented a systematic approach to commissioning of the complete compensator‐based IMRT planning and QA for the brass compensators manufactured by .decimal, Inc. Some model parameters for the beams modulated by the variable‐thickness compensators can only be associated with one compensator thickness. To intelligently choose that thickness for beam modeling, we empirically determined the most probable filter thickness occurring within the modulated portion of the compensators typically used clinically. We demonstrated that a set of relative output factors measured with the brass slab of most probable thickness (2 cm) differs from the traditionally used open field set, and leads to improved agreement between measurements and calculations, particularly for the larger field sizes. By iteratively adjusting the modifier scatter factor and filter density, the calculated effective attenuation of the flat filters was brought to within 2% of the ion chamber measurement for the clinically‐relevant range of filter thicknesses, depths and filed sizes. Beam hardening in Pinnacle provides for adequate PDD modeling beyond the depth of about 5 cm. Disagreement at shallower depth for the large field sizes is likely due to the algorithm's inability to account for the low‐energy scattered photons generated in the filter. The average composite ion chamber point dose error at isocenter for ten clinical plans was under 1%. A biplanar 3D diode dosimeter was calibrated and validated for use with the brass compensators. Its use in the clinic is particularly attractive because it easily generates, in one measurement set, dose comparisons at both the fraction and beam level. Overlaying dose profiles on the beam level would easily uncover any errors in compensator orientation which, in our experience, is the most frequently occurring potential error in compensator‐based IMRT.

## ACKNOWLEDGMENTS

This work was supported in part by a grant from .decimal Inc.
